# Contraction of Respiratory Viral Infection During air Travel: An Under-Recognized Health Risk for Athletes

**DOI:** 10.1186/s40798-024-00725-5

**Published:** 2024-05-22

**Authors:** Olli Ruuskanen, Henrik Dollner, Raakel Luoto, Maarit Valtonen, Olli J. Heinonen, Matti Waris

**Affiliations:** 1 Department of Paediatrics and Adolescent Medicine, Turku University Hospital and University of Turku, PL 52, 20521 Turku, Finland; 2 Department of Clinical and Molecular Medicine, Children’s Clinic, St. Olavs University Hospital, Norwegian University of Science and Technology, Trondheim, Norway; 3 Research Institute for Olympic Sports, Jyvaskyla, Finland; 4 Paavo Nurmi Centre and Unit for Health and Physical Activity, University of Turku, Turku, Finland; 5 Institute of Biomedicine, University of Turku and Department of Clinical Virology, Turku University Hospital, Kiinamyllynkatu 10, 20520 Turku, Finland

**Keywords:** Air travel, Aircraft, Respiratory virus, Infection, COVID-19, Athlete, Sport

## Abstract

Air travel has an important role in the spread of viral acute respiratory infections (ARIs). Aircraft offer an ideal setting for the transmission of ARI because of a closed environment, crowded conditions, and close-contact setting. Numerous studies have shown that influenza and COVID-19 spread readily in an aircraft with one virus-positive symptomatic or asymptomatic index case. The numbers of secondary cases differ markedly in different studies most probably because of the wide variation of the infectiousness of the infector as well as the susceptibility of the infectees. The primary risk factor is sitting within two rows of an infectious passenger. Elite athletes travel frequently and are thus prone to contracting an ARI during travel. It is anecdotally known in the sport and exercise medicine community that athletes often contract ARI during air travel. The degree to which athletes are infected in an aircraft by respiratory viruses is unclear. Two recent studies suggest that 8% of Team Finland members traveling to major winter sports events contracted the common cold most probably during air travel. Further prospective clinical studies with viral diagnostics are needed to understand the transmission dynamics and to develop effective and socially acceptable preventive measures during air travel.

## Introduction

An aircraft composes the major risk factors for a contraction of viral acute respiratory infection (ARI), fulfilling the “3Cs”: closed environment, crowded condition, and close-contact setting [1]. Pre-flight queueing at the security checkpoint, waiting at the airport, use of lavatory rooms and after the flight, queuing to exit the aircraft, and lining up at border entry are additional risk factors outside the aircraft. Furthermore, the use of common transportation, bus or train, before and after the flight increases the possibility of contracting an ARI [2–6]. Epidemiologic observations suggest that there are infectors on every flight. In two studies 17% and 21% of airline passengers reported symptoms of the common cold, making them potential spreaders of ARI [7, 8].

Frequent traveling to competitions and training camps is an essential part of an elite athlete’s life [9, 10]. The risk for an individual athlete to contract an ARI during air travel is real and familiar to sports physicians. Athletes traveling over more than five time zones have a two to threefold increased risk of illness [9]. In Norwegian cross-country skiers, the single greatest risk factor for infections was international air travel [10].

## Aircraft Cabin Environment

**Fig. 1:**
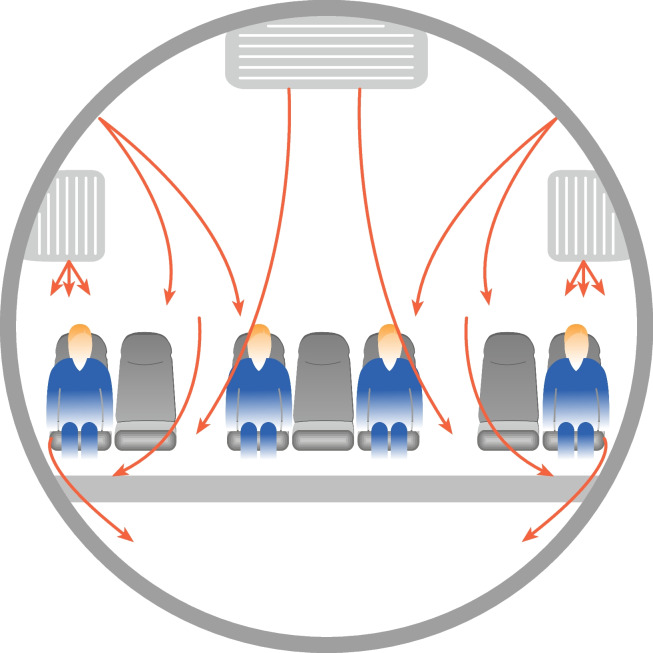
Airflow in an aircraft cabin (Airbus A320)

Aircraft cabin pressure, ventilation, humidity, and temperature are planned for passenger comfort and health. In the economy class the distance from a seat to the one in front or behind (seat pitch) is most often 81 cm and in the business class 160 cm. (Table 1 and Fig. 1). The aircraft cabin pressure is usually pressurized under 2500 m which is the threshold for increased risk of developing mountain sickness (shortness of breath, headache, lightheadedness, loss of appetite, dizziness) [11]. Half of the cabin circulating air comes from outside, the other half is recycled through high-efficiency particulate air (HEPA) filters [5]. The filters can capture particles sized down to 0.1 µm. Airspeed is typically < 1 m/s while cough induces an air speed of 2‒20 m/s [12, 13]. The concentration of CO_2_ during flight can reach 2000 ppm when a reading of 800 ppm or lower is indicative of good ventilation [14]. The relative humidity of cabin air is usually low due to outside air taken for recirculation and that with zero humidity. Low humidity is a major cause of complaints such as dry eyes and sore throat from passengers.

**Table 1:** Environmental factors in aircraft cabins affecting transmission of respiratory viruses

Passenger seat numbers up to 840 (Boeing 840)	
Duration of flight 2–4 h, maximum 18 h	
Temperature 21–25 °C	
Distances between a passenger in front or behind are most often 81 cm in the economy class	
Pressurized to an altitude of 1500–2438 m	
Air changes 20–30 times per hour	
Half of the air is recirculated	
Airflow compartmentalized into 4- to 7-seat rows	
Longitudinal airflow is minimal	
Air enters from overhead inlets and flows downwards through floor-level outlets	
HEPA filters used for air disinfection	
Humidity 10–25%

## Transmission of Respiratory Viral Infections

For decades respiratory viruses were mistakenly thought to transmit mainly through large respiratory droplets over short distances or touch [15–17]. Extensive research catalyzed by the COVID-19 pandemic has resulted in a paradigm shift suggesting that aerosol-based transmission may be the dominant route for all respiratory viruses [18, 19]. Aerosols are particles less than 100 µm in diameter. A person with a respiratory virus infection produces virus-laden aerosols during breathing. Importantly, speaking increases aerosol production by 35-fold and exercise by 132-fold [20]. The amount of viral shedding varies between different viruses and different SARS-CoV-2 variants. Some individuals may be superspreaders [21]. Virus-laden aerosols may spread everywhere in a confined space e.g., in the aircraft, and remain infectious for hours. Viral transmission occurs by direct inhalation of aerosols. The new WHO technical report abandons the division between droplets and aerosols, proposing a common term “infectious respiratory particles” which transmit “through the air” [19].

A spray of droplets and touch are the other possible routes of viral transmission. Droplets are particles more than 100 µm in diameter, and they spread only 0.2 m from a talking infectious person [22]. Transmission through touch is introduced by hands. It has been generally thought that the virus can be picked up by hand from highly touched surfaces e.g., door handles, desks, and elevator buttons and then the hand introduces the virus onto the mucosa of the nose and eyes. Spray and touch are now considered as possible routes of transmission but their role in real-life is not well documented [23, 24].

Close contact and the time spent in a crowded indoor space are the key determinants in the transmission of respiratory viral infections. According to the Centers for Disease Control and Prevention (CDC), close contact has occurred when a person is less than 1.8 m away from the other person for a cumulative total of 15 min or more over 24 h [25]. In addition, close contact requires a 2-way face-to-face conversation of ≥ 3 words [26]. After a 30-min interaction with a symptomatic child with ARI (mostly caused by rhinoviruses), 15% of the contacts were infected [27].

## Symptomatic and Asymptomatic Respiratory Viral Infections

Respiratory viral infections are the leading cause of acute illnesses. In 2019 the estimated incident cases of ARI reached more than 17 billion. The annual mean number was 2.25 episodes [28]. In one study on 6492 individuals in the USA, the weekly occurrence of ARI (sore throat, runny nose, cough, fever) was 4.4–5.7% [29]. In another study on 4102 individuals in Germany, the corresponding numbers were 2.7–8.2% [30]. On a single day in December in Sweden 29% of 232 adolescents had symptoms of respiratory infection but they were healthy enough to attend school [31]. It can be generalized that in adults symptomatic ARI incidence is about 5% per week during fall and wintertime.

The SARS-CoV-2 pandemic has demonstrated that a significant part of acute respiratory viral infections is asymptomatic. Several studies have searched for respiratory viruses in asymptomatic subjects [32]. Nasal swabs were collected biweekly from November until April from healthcare workers and seven samples (3.5%) of 200 asymptomatic subjects were virus-positive [33]. Nasopharyngeal swabs were collected from 2685 visitors to a New York City tourist attraction. A total of 6.2% of the samples were positive for a virus, 5.6% in the summer arm and 7.0% in the winter arm. Depending on the definition, 65‒97% of the infections were considered asymptomatic [34]. In a prospective follow-up study, 108 individuals were investigated by collecting weekly symptom diaries (n = 4550) and nasal swabs (n = 4506) for one year. In any given week 16% of the adults were virus-positive. Importantly, of the children less than five years of age 50% were virus-positive at any given time. In 44% of the viral detection, episodes were asymptomatic [35, 36]. The most common causative agents were rhinoviruses and seasonal coronaviruses out of 16 possible causative viruses [35, 37]. In a recent analysis of 14 studies, the pooled proportion of asymptomatic infection with the Omicron variant of SARS-CoV-2 was 25.5% [38].

## Respiratory Viruses at the Airport

Respiratory viruses have been detected frequently from touched surfaces. At two airports 15 (12%) viruses were detected in 130 surface samples; six seasonal coronaviruses, four rhinoviruses, four adenoviruses, and one influenza A virus [39, 40]**.** Chair handles, toys in the children’s playground, and hand-carried luggage trays were the most common virus-positive surfaces.

## Transmission of Respiratory Viral Infections Onboard

Contraction of a respiratory viral infection onboard is a result of many heterogeneous factors affecting viral transmission dynamics. The risk is highest during fall and winter when the viral infection rate in the surrounding community is highest [41, 42]. Many behavioural factors may enhance the risk of viral ARI like duration and proximity of the contact, symptoms of the infector e.g., sneezing and coughing, movement of the infectee and infector during the flight, and increased verbal interaction [41, 43, 44]. Immunobiological factors like the age of the infector, susceptibility of the infectee, viral load, and the degree of aerosol shedding as well as the respiratory intensity and infectivity of the virus affect the transmission. Also, cabin environmental factors affect the transmission including occupancy density, ventilation, and humidity [45]. Thus it is understandable that the number of secondary cases in different studies has ranged from 0 to 72% [4, 46, 47]. Most studies have focused on the spread of influenza and COVID-19. Furthermore, all studies carried out have several limitations. It is difficult to prove that a passenger was infected onboard, at an airport, or during ground transportation. Follow-up of all passengers for the incubation period (2–6 days) of respiratory viruses after the flight is difficult and incompletely carried out in many studies [7, 48].

### Influenza

A classic study in 1979 reported the spread of influenza A H3N2 among 54 passengers during a flight in Alaska [46]**.** Engine failure of the aircraft resulted in a three-hour ground delay with the ventilation system turned off and doors closed, and most passengers stayed on the aircraft. The infector was a young woman who 15 min after boarding became acutely ill with fever, chills, and severe cough. Influenza A was diagnosed serologically. Within 72 h, 72% of the passengers reported symptoms of ARI, influenza A H3N2 was cultured from eight passengers and 20 passengers had serologic evidence of influenza A.

The follow-up of 2165 passengers after possible exposure to influenza A virus (H1N1) pdm09 aboard an aircraft identified 163 secondary cases in 11 studies [2]. The secondary attack rate was 7.5%. Of the secondary cases 58% were seated at a greater distance than two rows from the index case [2]. Another analysis of 14 influenza A (H1N1) pdm09 inflight outbreaks revealed that the risk ratio for passengers seated within and beyond the two rows of the index case was 4.3 for laboratory-confirmed secondary cases [4]. The attack rate increased linearly with an increase of the product of the flight duration and the total infectivity of the index case.

### SARS-CoV-2

At the very beginning of the COVID-19 pandemic, the risk of transmission aboard aircraft was considered “nearly nonexistent” by the airline industry because there were flights with no secondary cases [47, 49, 50]. However, during the three years of the pandemic, it has become clear that air travel is one of the major risk factors for contracting SARS-CoV-2 infection although there is a high variability (0–62%) of secondary attack rates [51]. This is understandable because there is substantial heterogeneity in infectiousness. In some cases, superspreading may be an explanation for the marked spread of infection [21].

The risk of COVID-19 during 2020 was studied in a total of 177 airplanes departing from Wuhan city from January 2020 to March 2020 [52]**.** A total of 175 index cases were identified with 34 passengers considered to be infected on airplanes. The seats adjacent to the index case had an attack rate of 9.2%. A systematic review of the literature up to January 2021 included 18 studies on in-flight SARS-CoV-2 transmission with 130 unique flights [53]. A total of 2800 out of 19,729 passengers were traced and 272 index cases were reported with 64 secondary cases. The secondary attack rate varied between 0 to 8.2%.

Genomic evidence of in-flight transmission of COVID-19 has been shown in six studies [54–59]. On 1 July 2020, a 354-seat aircraft departed Dubai and landed in Perth Australia after a 10-h flight [59]**.** Within 95 passengers there was one symptomatic index case and the other index case developed symptoms within two days of arrival in Perth. During the 14-day quarantine 15 secondary cases were identified with an attack rate of 16%. Being seated within two rows of a primary case and spending more than an hour at the arrival airport were independent risk factors. Three cases in business class were infected despite having no contact with those in economy class. Whole genome sequencing revealed the circulation of the B.1480 variant in the 17 infected passengers.

The Omicron variant is markedly more transmissible than the earlier variants. In a recent study, the Omicron BA.1 variant was detected in aircraft wastewater [60]**.** The variant was then detected in 11 (20%) of the 56 passengers.

### Other Respiratory Viruses

Transmission of SARS-CoV-1 on aircraft is well documented. During one 3-h flight with 120 individuals including one SARS-CoV-1-positive index case, SARS was transmitted to 22 (18%) people [61]. In contrast, contact tracing of 21 cases of MERS-CoV on 31 flights revealed no cases of in-flight transmission [62].

Rhinoviruses and non-COVID coronaviruses (“common cold coronaviruses”) are the most common respiratory viruses causing the common cold, being responsible for 60–70% of the cases [63]. It is highly probable that in nearly every flight there are rhinovirus- or coronavirus-positive individuals who are potential infectors [64, 65]. In one study respiratory symptoms were reported by 17% of 15,976 airline travelers arriving at Christchurch International Airport, New Zealand during influenza season [65]. Respiratory viruses were identified in 342 (26%) of 1313 combined throat and nasal swab samples. Rhinoviruses (10%) and enteroviruses (6%) were the most common. Surprisingly, there are no studies on the spread of these viruses onboard*.*

Guidelines for contact tracing have traditionally recommended searching passengers who were seated during the flight within 2 rows of the infector i.e. within the close contact definition of 1.8 m. However, during the COVID-19 pandemic it became clear that the 2-row rule may fail to detect many infected cases. Even passengers in business class may contract ARI without a symptomatic index case in the business class [59].

## Respiratory Viral Infections in Athletes Associated with Air Travel

There is very little information on health risks in traveling athletes. In one study 259 elite rugby players from eight teams were followed daily over the 16-week competition period with frequent flying [9]. International travel to a foreign country with a > 5 time zone difference was associated with a 2–3 times increase in the incidence of respiratory tract illness. No detailed information on the clinical manifestations or etiology of illnesses was reported.

In our study on the 2018 Winter Olympic Games (PyeongChang), three members of Team Finland (n = 122) developed respiratory symptoms (1 respiratory syncytial virus A, 1 influenza B virus, and 1 respiratory syncytial virus B) onboard during a 9-h flight to Seoul [66]. Two team members with coronavirus OC43 were most probably traveling during the incubation period of the infection [48]. Thus at least 4.5% of the team was virus-positive during the flight. During the first four days after arrival, six (5% of the team members) more virologically defined ARIs caused by rhinoviruses, RSV A, coronavirus OC43, and influenza type B were detected suggesting contraction during the flight [66].

A prospective study on respiratory viral infections among 62 Team Finland participants was carried out during the Nordic World Ski Championships 2019 [67]. Nasal swabs were taken at the onset of a symptom and on days 1, 7, and 13 during the competition, which lasted for 14 days. After the flight to Seefeld (day one) seven subjects were virus-positive. Nine further subjects were identified as virus-positive during the first five days of their stay suggesting contraction during air travel. Six different respiratory viruses were detected in the team. The obvious transmission rate of viral ARI during air travel was 15%.

During the COVID-19 pandemic prevention procedures, we studied respiratory viral infections among Team Finland participants (73 athletes and 110 staff members) during the 2021 Oberstdorf World Ski Championships and the 2022 Beijing Olympic Games. Only six cases of symptomatic ARI were found. Five non-SARS-CoV-2 infections were detected at the site caused by rhinoviruses (2), respiratory syncytial virus (1), metapneumovirus (1), and coronavirus 229E (1) [68]. All infections were identified within three days after arrival suggesting possible contraction during air travel, and in any case being infectious onboard.

## Prevention Strategies for Athletes During Air Travel

Influenza A, influenza B, COVID-19, and respiratory syncytial virus infection can be prevented by vaccination. Interestingly, in a study on 13,865 patients with virus-associated lower respiratory tract infection and 227,887 matched controls pneumococcal conjugate vaccination was associated with moderate protection against virus-associated infections [69]. Vaccination should be actively offered to elite athletes. It is worth stressing that influenza virus transmission can also be effectively prevented by antiviral (e.g. oseltamivir or baloxavir) treatment [70, 71].

An athlete should postpone travel if suffering from ARI to avoid further transmission during travel. This is a difficult option because mild to moderate ARI is not generally considered a reason not to travel. Passengers exhibiting symptoms of ARI should be advised at least to wear a mask during the entire air travel time [15].

Potentially effective prevention strategies for all viral ARIs were developed during the COVID-19 pandemic. The universal wearing of masks, school and restaurant closures, travel restrictions, a ban on mass gatherings, and other physical exclusion measures also dramatically reduced the occurrence of non-COVID respiratory viral infections in the community, especially influenza and respiratory syncytial virus infections [72]. Due to the public health preventive measures, personal countermeasures, and traveling by chartered flights, travel-associated respiratory viral infections were observed to have dropped to 3% in Team Finland upon arrival at the 2021 World Ski Championships and the 2022 Beijing Olympic Winter Games; this was a much lower percentage when compared to the 8% and 15% in our two earlier corresponding studies [66–68].

**Fig. 2:**
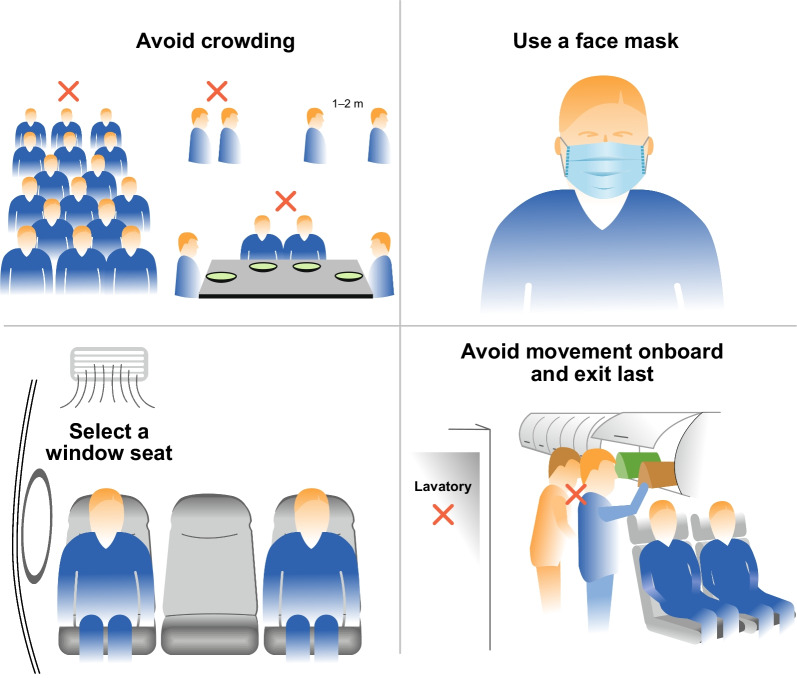
Simple infection prevention measures for elite athletes during air travel

It could be beneficial for athletes to maintain some of the prevention measures after the pandemic [73]. It should, however, be noted that the efficacy of these measures during air travel is not well documented in real-life studies. The well-known measures to be considered are shown in Table 2 and Fig. 2. Several studies suggest that in aircraft a well-fitted FFP2/N95 mask, although uncomfortable, may decrease viral transmission by 65‒90% [72, 74–77]. However, face masks alone may not be sufficient to prevent transmission [78]*.* Despite mandatory mask use and pre-departure testing on two flights, four passengers transmitted SARS-CoV-2 infection to at least 21 (6%) of the 345 passengers [58]. Hence, multiple measures may be needed. Traveling by public transportation before and after air travel increases the risk of transmission and airport stays should be kept as short as possible [79]*.*

**Table 2:** Potential prevention strategies for athletes to reduce the risk of contracting a viral respiratory infection during air-travel

*When reaching and leaving the airports*	
Avoid using common transportation i.e. trains or buses	
*At the airports*	
Minimise the time at the airports	
Use a face mask when arriving at the airport and until leaving the airport	
Minimise movement at the airport	
Avoid crowding e.g. restaurants, shopping	
Minimise lavatory use	
Use an alcohol-based hand sanitiser after leaving the airports	
*On the aircraft*	
Passengers with respiratory symptoms should be supplied with a mask and seated, if possible, separately	
Board and deplane first or last to minimise crowding during loading/unloading of the overhead luggage compartment	
Select a window seat, avoid an aisle seat	
*Turn on the gasper above your seat*	
Physical distancing when feasible during boarding and deplaning	
Use an alcohol-based sanitiser after boarding and during the flight	
Avoid movement during the flight	
Change seats, if possible, if a nearby passenger is coughing	
Minimise food and drink services to avoid contact and to reduce the time necessary to be unmasked	
If possible, do not use the lavatories	
Avoid high touchpoint surfaces like tray tables, armrests, door knobs, and toilet flush buttons

In the aircraft, the clustering of passengers while waiting for others to stow their luggage and take their seats increases exposure to infection [80]*.* Increasing evidence supports the view that a window seat is the safest seat because it is associated with the smallest number of contacts during the flight. In a study on 10 transcontinental flights, the 10-member research team recorded the movements of passengers [43]***.*** Passengers in window seats had 12 contacts compared to 58 and 64 in the middle and aisle seats, respectively. In a model study, vacant middle seats reduced SARS-CoV-2 infections by 37% [81]**.** The authors concluded that, if the aircraft is not fully occupied, the middle seat should be kept empty. Changing seats is recommended if a passenger within two rows is coughing [82]*.* In a simulation study with a cough generator, the passenger in front of a coughing passenger had a four times higher risk than the other passengers [83]. It is advisable to turn on the air outlet (gasper) situated above each passenger seat; this can decrease cough and talk droplets and reduce the infection risk of the passenger in front of an infectious person [5, 83]*.* Frequent use of hand sanitizers and avoiding high-touch surfaces is encouraged although their efficacy in real life has not been studied [84]. Moving in an airplane may markedly increase the risk of viral transmission. The most common movement is waiting for, using, or exiting a lavatory and checking the overhead luggage compartment [43]*.* During an 81-min domestic flight, an aircrew member’s movement while serving food and drinks likely transmitted the Delta variant SARS-CoV-2 to 11 (7.6%) of 145 contacts [85].

## Conclusions

Transmission of viral ARIs during air travel and especially in flight is well established. Current evidence suggests that approximately 5–10% of athletes could contraction viral ARI during wintertime air travel. A febrile ARI may ruin the athlete's participation in a major competition. The risk for viral ARI transmission can be reduced during travel by well-known multiple intervention measures including the use of a mask, maintaining physical distance as much as possible, and enhancing hand hygiene. The prevention strategies selected should be well accepted by the athletes and not induce social or psychological stress.

## Data Availability

Not applicable.

## Abbreviations

ARI: Acute respiratory infection
HEPA: High-efficiency particulate air filters
WHO: World Health Organization
